# Supplementary material for: Predicting visual function by interpreting a neuronal wiring diagram
Source: Nature. 2024 Oct 2;634(8032):113–23. doi: 10.1038/s41586-024-07953-5 (PMC11446822; doi:10.1038/s41586-024-07953-5)
Supplement: Supplementary file 6 — CRF and ERF predictions for individual TmY4 and TmY9 cells. Analogous to Supplementary Data 3, but for TmY target types. Shown are the top four monosynaptic pathways, the strongest pathway passing through each of the top ten intermediary types (ranking from Extended Data Fig. 7), and the trisynaptic pathway Tm1–TmY–Dm3–TmY (see the section entitled Prediction of spatial normalization). [file 41586_2024_7953_MOESM6_ESM.zip › DataS4/TmY9q/720575940617608665.pdf]

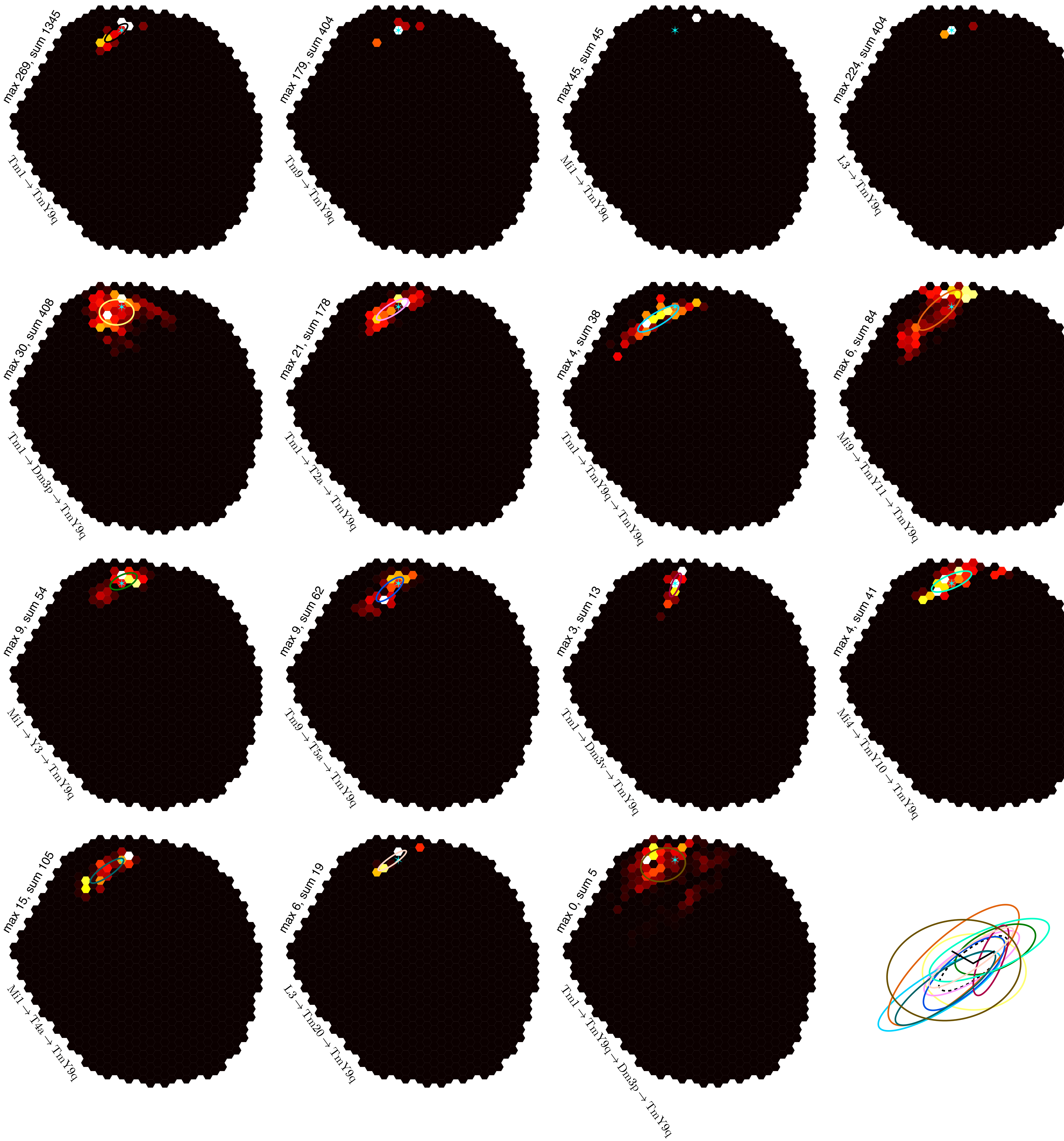

max 269, sum 1345
Tm1 → TmY9q
max 179, sum 404
Tm9 → TmY9q
max 45, sum 45
Mi1 → TmY9q
max 224, sum 404
L3 → TmY9q
max 30, sum 408
Tm1 → Dm3p → TmY9q
max 21, sum 178
Tm1 → T2a → TmY9q
max 4, sum 38
Tm1 → TmY9q → TmY9q
max 6, sum 84
Mi9 → TmY11 → TmY9q
max 9, sum 54
Mi1 → Y3 → TmY9q
max 9, sum 62
Tm9 → T5a → TmY9q
max 3, sum 13
Tm1 → Dm3v → TmY9q
max 4, sum 41
Mi4 → TmY10 → TmY9q
max 15, sum 105
Mi1 → T4a → TmY9q
max 6, sum 19
L3 → Tm20 → TmY9q
max 0, sum 5
Tm1 → TmY9q → Dm3p → TmY9q
